# Global Health Is Local Health: A Multidisciplinary Perspective of COVID-19

**DOI:** 10.31486/toj.20.0059

**Published:** 2020

**Authors:** Angelica Hinchman, Diab Ali, Bailey W. Goodwin, Monica Gillie, Jacob Boudreaux, Yvens Laborde

**Affiliations:** ^1^ The University of Queensland Faculty of Medicine, Ochsner Clinical School, New Orleans, LA; ^2^ Medical Director, Global Health Education, Medical Director, Public Health, Ochsner Clinic Foundation, New Orleans, LA

## INTRODUCTION

In December 2019, the novel coronavirus—severe acute respiratory syndrome coronavirus 2 (SARS-CoV-2)—likely originated from a wet animal wholesale market in Wuhan, China.^1^ Originating from 5 hospitalizations for respiratory illness in the Hubei Province on December 29, 2019, infections quickly erupted to 571 confirmed cases by January 22, 2020 in 25 provinces throughout China and to 1,975 cases by January 25, 2020.^1-3^

The affordability and availability of modern travel, crowded airports, and the recycled air of planes aided in unprecedented geographic reach and rapid escalation of viral transmission.^4^ Globalization, in combination with the prolonged incubation of COVID-19, its lengthy survival time on surfaces, and the extended period of viral shedding in infected persons, enhanced the virus's largely undetected proliferation. By January 30, 90 additional cases were reported beyond China's borders in Taiwan, Thailand, Vietnam, Malaysia, Nepal, Sri Lanka, Cambodia, Japan, Singapore, South Korea, United Arab Emirates, United States, Philippines, India, Australia, Canada, Finland, France, and Germany.^1^ The virus spread beyond local and national borders. What was previously a vague and distant global health problem quickly became a local public health concern everywhere.^5^

By the end of March 2020, New Orleans, LA, had emerged as the city with the highest fatality rate per capita in the United States.^6^ This fatality rate disproportionately affected African Americans who accounted for 70% of coronavirus deaths in Louisiana, while making up only 32% of the state's population.^7^ The heavy burden of noncommunicable disease in Louisiana, compounded by recent social events in the city of New Orleans, including the Carnival season, catalyzed the rapid transmission of SARS-CoV-2 throughout the state.^6^

In this paper, we review the multidisciplinary impacts of the COVID-19 pandemic across the healthcare system. From a local focus on New Orleans to a global perspective, we relate how rapidly changing healthcare policy, evolving use of technology, and social media dynamics played roles in perception and response to the pandemic. We reflect on the perspectives of evolving national health policy, public health demands, impact on mental health, strain on primary and emergency care, and the emergence of telehealth on a global and local scale.

## UNITED STATES HEALTH POLICY AND INFORMATION SHARING

**Figure 1.**
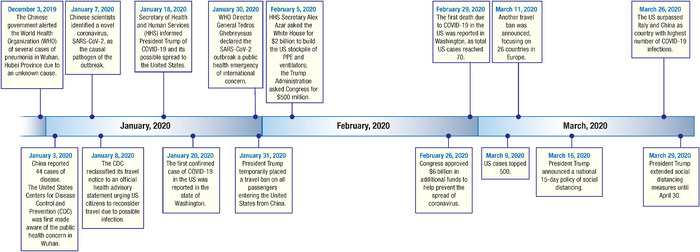
**Timeline of US response to COVID-19 pandemic.**^8^^-^^10^ PPE, personal protective equipment; SARS-CoV-2, severe acute respiratory syndrome coronavirus 2.

The rapid escalation from the outbreak in Wuhan, Hubei Province, China, to global pandemic in a matter of 30 days presented a challenge for the coordination of a federal public health response in the United States. The federal government's response during the early days after the World Health Organization alert on December 31, 2019 consisted of evacuating American diplomats from Wuhan, banning air travel from China, and preparing to repatriate Americans from abroad while managing stirring fears at home (Figure 1).^8-10^

The government's facilitation of communication between healthcare experts and the public during an emerging pandemic amplified an existing challenge in public health: finding a balance between informing the public of imminent health threats while preventing increased anxiety and panic in response to elevated risk perceptions.^11^ As US government officials struggled to keep up with the rapidly evolving data on COVID-19, a disjointed public health policy response resulted, leading to public distrust, panic, and polarized perceptions of the disease.

As in China, delays in information sharing and guidelines by the US government created a window of uncertainty, providing the opportunity for social media platforms to assert themselves as primary news sources for the American people, resulting in rumors and misinformation being spread within the United States via social media posts and the sharing of obscure news outlets.^12,13^ Content-shaping algorithms that personalize the user experience, popular on websites such as Facebook, compounded the cycling of misinformation, enabling public confusion, anxiety, and mistrust of the government. One outcome was the panic buying of toilet paper, hand sanitizer, antimicrobial wipes, and other goods during the early days of the COVID-19 outbreak.^12-14^

Public confusion was amplified by inconsistent information shared by US government representatives. On March 16, President Trump announced a national 15-day policy of social distancing.^15^ Six days later, the President tweeted that social distancing may be “worse than the problem itself.”^16^ On April 3, although the President echoed recommendations by the US Centers for Disease Control and Prevention (CDC) for Americans to don cloth face coverings while in public, he emphasized that these recommendations were temporary and voluntary and stated that he would not wear a mask himself.^17^ Such communications led to the emergence of competing narratives surrounding how people should best protect themselves and others from the virus based on a patchwork of guideline interpretations. The contradictory information from government officials demonstrated the importance of having coordinated public health policy information and responses.

## HEALTH AND SOCIAL DISPARITIES

**Figure 2.**
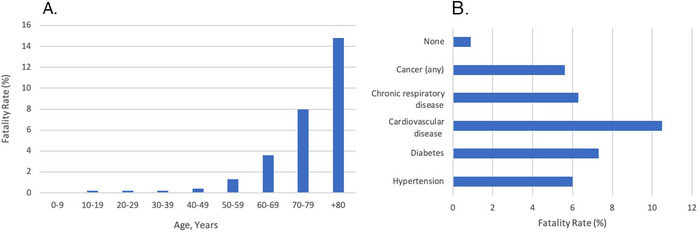
**Fatality rate by age bracket (A) and comorbid conditions and associated fatality rates (B).**^18^

The majority of COVID-19 confirmed cases appeared to occur in patients 30 to 60 years of age (77.8%), but the population group with the highest mortality are those ≥60 years old.^18^ Preliminary findings from the China CDC show a positive correlation between age and fatality (Figure 2).^18^ Among 13,909 cases, confirmed COVID-19 positive patients ≥60 years of age had a 4.06% mortality rate.^18^

Age is not the only contributing factor for increased mortality; preexisting comorbidities including hypertension, diabetes, cardiovascular disease, chronic respiratory disease, and all types of cancer increase the risk of fatality compared to having no comorbid conditions at all.^18^ However, these factors are not mutually exclusive as older patients are more likely to have one or more comorbid conditions.

**Figure 3.**
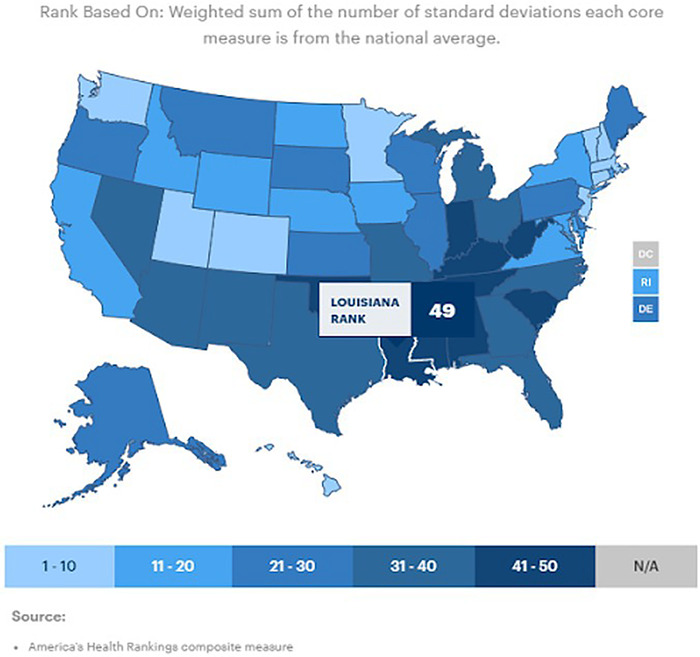
**National map of health outcome rankings by state.**^19^ (Republished with permission ©2019 United Health Foundation. All rights reserved.)

The state of Louisiana is particularly at risk given the prevalence of comorbidities in the population. In the most recent census (2019), Louisiana ranked 49th in overall health outcomes based on behavior, policy, clinical care, community, and environment (Figure 3).^19^ In reports focused on particular conditions, 39% of adults were reported as having hypertension, 14.1% with diabetes, 6.8% with chronic obstructive pulmonary disease, and 6% with heart disease.^20-23^ The prevalence of these comorbid conditions makes the population of Louisiana as a whole susceptible to poor health outcomes.

**Figure 4.**
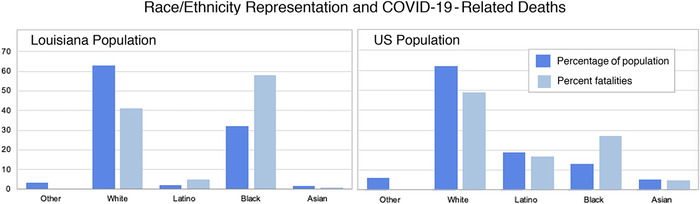
**Race/ethnicity representation in the United States and in Louisiana and the percentages of COVID-19–related deaths through May 8, 2020.**^7^

In addition to the effect of comorbid conditions, preliminary data revealed race as a contributing factor in COVID-19 cases and mortality. In Louisiana as of early May, African Americans accounted for approximately 60% of COVID-19–associated fatalities (Figure 4).^7,24^ Statewide data showed that 40% of deaths occurred in Orleans Parish, where roughly 60% of the population is African American.^7,25^ Consolidated CDC data from 14 states from the month of March showed that 33% of hospitalized, COVID-19–confirmed patients were black, while this group made up only 18% of the surrounding population.^26^ In New York City, both African American and Hispanic COVID-19–related death rates outnumbered those of whites or Asians.^26^

Meanwhile, the American Public Media Research Lab independently began collecting racial breakdown data from the District of Columbia and 36 states and found alarming evidence of a 150% increased likelihood of COVID-19 fatality among black patients compared to white, Hispanic, or Asian patients.^7^ An important note is that the collective data reporting on COVID-19 are incomplete, as states are only reporting the racial breakdown on a portion of recorded deaths.

This racial disparity is thought to be attributable to a combination of socioeconomic factors, including a long history of structural injustice and oppression in the United States that has resulted in social inequity, higher rates of chronic conditions, poverty, and unemployment among African Americans.^27^ This sentiment is reflected in data demonstrating that African Americans aged 18 to 49 years are twice more likely to die from heart disease, and, on average, develop heart disease at a younger age than white Americans. This statistic is in addition to documented higher rates of hypertension, diabetes, cerebrovascular disease, asthma, and obesity in African American communities.^27^

Furthermore, minority populations are more likely to work in the so-called essential labor sector as workers in healthcare, transportation, government, and food supply.^25^ One in 5 African Americans and 1 in 6 Hispanics have a job that does not allow them to stay at home and social distance.^25,28^ Minority populations’ ability to social distance is also impacted by the higher likelihood of living in multigenerational households and densely packed living situations.^28^ Finally, African Americans are more likely to report financial barriers to accessing healthcare.^27^ Financial barriers may delay presentation, allowing case severity to advance and increasing the likelihood of intensive care unit (ICU) admission. The racial bias, stereotyping, and prejudice known to affect quality of care by healthcare providers, as well as patient experience in the healthcare system, are also contributing factors.^29^

To further explore these racial and demographic disparities, more comprehensive research and data are needed to guide population-specific interventions and health policy. Without these data, health officials and lawmakers will not be able to adequately address health inequities or understand the scope of the disparities.

## EMERGENCY MEDICINE

Emergency departments (EDs) and urgent care centers across the world are classically on the frontline of disaster and are where the first encounters with patients generally occur. As rumors mounted and anxiety built in the absence of communication from national leaders, EDs were flooded by patients with COVID-19–related complaints, reaching a total of 1,449,351 visits in the United States and peaking at 106,972 visits in 1 week.^30^ With the influx of patients rushing to hospitals for care, resources were reallocated to address the dramatic increase in patient volume and to mitigate transmission within these facilities.

Healthcare institutions across the globe prioritized frontline emergency and intensive care departments to directly address mounting patient demands. Resources, including personnel, were shuffled within systems, and elective and nonemergent procedures were canceled. These steps had the positive effects of distancing healthy patients from disease-laden hospitals, permitting the conversion of unused space to additional ICU capacity, and conserving personal protective equipment (PPE), but the resource reallocation also left a void in the care of thousands of patients.^31^

As crowded waiting rooms devolved into vectors for viral spread with the surge of patient encounters, protective measures became necessary to decrease transmission among waiting patients. One hospital in Taiwan implemented buffer areas to segregate patients based on risk. Patients with fever and respiratory tract symptoms were categorized into high, intermediate, or undetermined risk groups and allocated to separate areas to minimize the potential spread of COVID-19 throughout the hospital.^32^ Patients were managed in these designated areas and transferred to isolation rooms for the collection of nasopharyngeal swabs. By establishing this protocol, the hospital was able to achieve a positive predictive value of 16.7% for COVID-19 infection in the high-risk area of the buffer zone.^32^ However, strategies such as these are hindered by time availability, lack of well-established protocols, and limited accessibility to PPE, further compounded by significant drops in PPE production in China.^33^

Institutions compensated for the increase of patient needs by establishing call-in telephone lines to mitigate the brunt of public panic and minimize the burden on the ED. Strategies evolved to keep healthier patients away from hospitals through the establishment of large-scale communication lines that encouraged home management of mild symptoms to limit transmission within healthcare institutions.^34^ Ochsner Health established a nurse- and medical student–run call center to triage sick patients outside of the ED that eventually evolved into a platform for symptom monitoring of COVID-19–positive patients from home.^35^ Integrating this call center into Ochsner's COVID-19 response plan enabled ongoing resource-sparing medical contact with a large patient population at one of the major hotspots for infection in the United States.

Despite the implementation of new hospital protocols in response to the COVID-19 pandemic, the public's fear of waiting rooms as vectors for disease swelled, and ED attendance began to drop while the percentage of COVID-19–associated illness continued to rise.^30^ Noninfected patients began avoiding the ED out of fear of contracting the virus. As a result, care was delayed for some patients with potential health emergencies, further contributing to the escalating mortality observed in the United States.^36^

Hospitals in other parts of the world have reported consequences of complex COVID-19 protocols. A hospital in Hong Kong, China, has documented evidence of delayed primary percutaneous coronary intervention treatment of ST-segment-elevation myocardial infarction, not only because of delays in seeking care but also because of institutional delays caused by new COVID-19 safety protocols.^37^ Although necessary to limit the transmission of disease, some emergency and triage adaptations have resulted in unforeseen consequences to patient care.

## PRIMARY CARE

New Zealand and Denmark had some of the earliest and most aggressive responses to the COVID-19 pandemic. New Zealand began mandatory quarantines for all visitors on March 15, one of the strictest policies in the world at the time, even though only 6 cases had been documented nationwide. Just 10 days later, New Zealand instituted a complete countrywide lockdown, including a moratorium on domestic travel. Under these level 4 restrictions, grocery stores, pharmacies, hospitals, and gas stations were the only commerce allowed; vehicle travel was restricted; and social interaction was limited to within households.^38^ Denmark, which already has one of the world's highest functioning healthcare systems, had more than 7 times as many beds with ventilators as it had patients hospitalized with critical COVID-19 symptoms.^39^ Additionally, because of the universal healthcare model in Denmark, all treatment, including testing, was without cost to those who needed it.

In contrast, the primary care system in the United States lacks a standardized healthcare unit. Public and private outpatient practices vary in size and ownership, creating hurdles to identifying the problems COVID-19 presented to primary care.^40^ The Primary Care Collaborative (PCC) began conducting weekly surveys to try to identify the issues primary care workers were facing. On March 19, 2020, the PCC published results from a survey of more than 500 physicians, nurses, nurse practitioners, and physician assistants working in the primary care sector.^41^ Survey responses indicated limited capacity for testing in the outpatient setting, which was compounded by the stress of addressing an abundance of patient concerns, challenges in limiting the exposure of healthy patients, and a shortage of PPE for both staff and patients.^41^ As the surveys continued in subsequent weeks, the stressors shifted to limiting routine visits and clinic staff absences because of illness or isolation.^42^ Despite patients being receptive to telehealth visits, 70% of primary care workers who responded to the survey did not work at a practice that offered e-visits.^42^ Interestingly, this number decreased to 50% by the next week, highlighting the rapid expansion of telehealth services.^43^ By week 5, more than 2,600 primary care workers from all 50 states had responded to the survey, and the major issues impacting access to care were identified as disparities among racial minorities, patients with low income, and patients with no internet access.^44^ The adaptations to minimize in-person appointments created new challenges for treating patients with preexisting conditions and training patients and physicians in a new way of practicing medicine. The week 8 survey showed that 38% of responders felt that patients avoiding or delaying care until after the pandemic will lead to non–COVID-19 deaths, and 60% believed that patients will experience avoidable illness.^45^ In England, primary care physicians suspended routine checks for all patients >75 years nationwide.^46,47^ Similarly, primary care physicians in New Orleans limited in-person visits and moved routine checks to telemedicine when possible. As a result, clinicians are having to learn new skills, platforms, and ways to care for patients.^48^

## IMPACT OF COVID-19 ON MENTAL HEALTH

From a healthcare standpoint, pandemics cause a predictable surge in demand because of the infectious agent itself and because of the associated mass anxiety. The unfamiliar and mysterious nature of an invisible agent makes it seem “powerful, evil, and imperceptible.”^49^ Further, as misinformation rapidly spread, the pandemic had a profound behavioral impact, resulting in avoidance or anger, scapegoating, disruption of work-life balance, restricted activities, and substance use.^50^ Factors such as the deaths of those seen as vulnerable, inadequate resources, paranoia, conspiracy theories, loss of faith in leaders and institutions, and the restriction of civil liberties all may have unforeseen impacts on downstream societal perception and social participation**.**^51,52^

In New Orleans, the compounded fears of infection, isolation, and quarantine, as well as resource shortages and scarcity, contributed to a heavy cognitive-emotional burden. Vulnerable populations requiring additional mental health considerations include migrants and refugees, people with cognitive or mobility impairments, the disadvantaged and homeless, children and adolescents, pregnant and postpartum women, and people who depend on systems of care.^50^ People with preexisting psychiatric disorders, including those with addiction and substance abuse issues, are especially vulnerable, not only to the uncertainty associated with COVID-19 but also to the potential to be more stigmatized and marginalized compared to their already problematic acceptance.^53^ At the end of March 2020, market research by Nielsen demonstrated a 55% increase in alcoholic beverage sales compared to the prior year, but only 8% of Americans reported an increase in alcohol or substance use intake.^54,55^

Despite the predictable increase in the need for mental health support, resources were reallocated to support primary care and hospital settings to directly address COVID-19 infection, leaving mental health services insufficiently prioritized in New Orleans. The New Orleans National Alliance on Mental Illness (NAMI) chapter had to suspend all new patient intakes, psychosocial rehabilitation, community support, and education programs in the early stages of the pandemic with scarce virtual options to take their place. A few New Orleans phone and virtual support groups have become available, including the #GetYaMindRight Virtual Support Group hosted by the Institute of Women & Ethnic Studies,^56^ the NAMI New Orleans–associated Survivors of Suicide Loss virtual support group,^57^ and the Louisiana Department of Health Office of Behavioral Health Keep Calm Through COVID crisis phone line providing mental health and substance abuse counseling services.^58^ However, these very few mental health resources adapted to address pandemic-relevant mental health may be underprepared for the compounding roles of providing specialized care to those with preexisting mental health disorders, substituting for preestablished community support groups, and handling pandemic-related psychiatric problems. Both in New Orleans and worldwide, substantial increases in anxiety and mood disorders, substance use, domestic violence, and child abuse appear to be likely as schools remain closed. The downstream effects of this mental health emergency are difficult to predict.

## TELEMEDICINE: ADAPTING A SOLUTION

Telemedicine was initially developed as a tool to address resource-limited populations that lacked accessibility to healthcare centers.^59^ It has increasingly become a focus of research in all health sectors globally, with the original emphasis placed on its use in psychotherapy and mental health services.^59^ Prior to the COVID-19 pandemic, telehealth was not recognized or established as an integral part of the American healthcare system and was accessible to only slightly more than 50 institutions across the country.^60^ Since the beginning of the COVID-19 pandemic, the needs for isolating patients, limiting exposure, providing medical maintenance, and delivering essential care have highlighted telemedicine as a critically important solution for healthcare delivery. In addition to conserving resources, providing virtual care through telemedicine allows physicians to assess infected patients and patients under investigation without risking exposure to the practitioner, support staff, and other patients. As the supply of PPE, medical staff, and hospital beds dwindled, the importance of effective, resource-conserving measures shined a spotlight on telemedicine. Now, more than ever, the global need for resource-sparing healthcare options is reflected on the local level.

The United States has been slow to incorporate telehealth into its healthcare systems, unlike other countries such as China.^61^ However, sudden demands for a platform that enabled social distancing–compliant healthcare access accelerated the adoption of telehealth in the United States. The COVID-19 Emergency Declaration^62^ allowed for enactment of an 1135 waiver under the Social Security Act to expand Medicare coverage to patients seen by video visit for any purpose.^63^ The 1135 waiver also allows for the prescription via telepsychiatry service of schedule II to V controlled substances that previously required an in-person visit, thus giving patients covered by Medicare access to medications without the risk of physical exposure in the office.^64^ This waiver bypasses major barriers to care, including limitation of services, range of treatment, and state-specific restrictions in licensing that stunted previous government telemedicine incentives. However, a gap remains for those covered by private insurance or Medicaid, for whom this waver does not apply to date.^64^

In New Orleans, telemedicine programs were adapted to address the needs of the pandemic. Patients in high-risk demographic groups were targeted specifically for remote visits so that they could avoid going into clinics and hospitals. The majority of these targeted patients had low socioeconomic status, high comorbidities, and low technology literacy, and they were older in age. While telehealth options circumvent classic socioeconomic barriers to care, such as transportation and financial restraints, new barriers such as the inability to be trained on and navigate the telemedicine system, lack of access to smart devices, and inadequate wireless internet connections are obstacles in converting to a virtual visit system. Inequities to accessing telemedicine services will need to be addressed in future health policies.

## DISCUSSION

### Social Media as a Tool for Public Health

Existing social factors—such as psychological, cultural, health, and socioeconomic status—collectively contribute to the public perception and interpretation of risk in a pandemic. Pandemic communications must balance public engagement to optimize a unified capacity for citizens to practice precautions that reduce transmission while also minimizing panic.

A 2016 article analyzing the role of UK Twitter in regard to information sharing during the 2009 H1N1 pandemic found that the largest category of information users interacted with were resource information tweets that shared links or were descriptive in nature.^65^ In this category, news sources had significantly more engagement than health authorities. News publications have the power to sway public perception through tone and recontextualization of the information being shared.^65^

Early sharing of information from public health officials may establish health authorities as the primary resource regarding health information, reduce public speculation and distrust, moderate public paranoia and anxiety, and unify public involvement in health initiatives such as social isolation. Increasing public health awareness by providing information and promoting guidelines could facilitate compliance with government mandates. One example is to develop a list of recommended items and suggested quantities for a 2-week quarantine per household. This public health promotion could be extended to grocery stores, with the recommendation to assemble kits of items to minimize shoppers’ time in public settings. Overall, a prompt and aggressive approach to information sharing by public health officials could ease public anxiety and enable an organized public response to improve health outcomes in a pandemic.

### Effects on Medical Students

The COVID-19 pandemic has affected all healthcare practitioners, including students. Medical students across the United States in their final 2 years of schooling, primarily working in the clinical setting, had their studies suspended to minimize their exposure to the coronavirus.^66^ These alterations may have a lasting impact on their careers. Removal from clinical settings, suspension of away rotations, and canceled board examinations left many third- and fourth-year students without the opportunity to meet the standard requirements of the National Resident Matching Program to secure an intern placement for the next year.^66^

At the Ochsner Clinical School in New Orleans, as at several other US medical schools, third- and fourth-year students refused to be sidelined. Despite a 2-week suspension of studies that removed students from their clinical rotations, approximately 151 students volunteered during their reallocated vacation time to assist in the response to the pandemic.^35^ Students volunteered at call centers, contributed to the development of Louisiana's first rapid testing center, and acted as family liaisons/advocates to take some of the load off of physicians and nurses who typically fill these roles, thereby allowing them to focus on direct patient care.^35^

### Supporting Public Mental Health

The need for public mental health support has been critically demonstrated during the COVID-19 pandemic, revealing weaknesses in both public and private healthcare systems and the need for improved mechanisms for basic care and refill/delivery of essential psychiatric medicines. Mental illness, already a barrier to traditional care because of its nature, was exacerbated by the isolation, anxiety, and compounded stigmas of the pandemic. The literature supports using a system of stepped care in treatment that involves the universal offering of effective, resource-conservative treatment and then advancing care as necessary based on individual patient needs.^67^ Given the capabilities of telehealth, virtual platforms could be used for psychiatric telehealth consults and group visits in resource-limited settings.

Even if these recommended changes are implemented, the healthcare system will likely remain insufficient unless community support is also strengthened. The social distancing measures of the COVID-19 pandemic resulted in a lack of programs that provided community support, rehabilitation, and therapeutic services. In conjunction with comprehensive telehealth programs, social media could be used to connect people to evidence-based mental health resources and healthy practices. These platforms could also integrate check-in functions; promote collective resilience in the face of community stressors; and improve systems of mental health surveillance, reporting, and intervention for the anticipated increase in domestic violence and child abuse during the COVID-19 pandemic. Further, nontraditional groups could be trained to provide psychological first aid, reinforcing the public's ability to check in with one another and provide effective support. These nontraditional groups can include community populations that have been well documented in studies of task-shifting in the global mental health literature (teachers, religious leaders, barbers) or could be those involved in healthcare (medical students, medical assistants, pharmacists, physical therapists). Training an expanded range of multidisciplinary healthcare team members in psychological first aid could also help address the mental health burden these providers have as vulnerable populations themselves. COVID-19 illuminated the need for increased understanding of the impact of collective community stressors on healthcare workers, as well as programs dedicated to optimizing the mental health of providers susceptible to both direct and vicarious trauma.

### Telemedicine

Telemedicine rapidly expanded to meet the demand of the COVID-19 pandemic. However, many obstacles to care still exist, including health access inequity. Patients without smart devices or internet access are unable to receive comprehensive, virtual care. This inequity is compounded by lagging health policy that historically has and continues to funnel funding for telemedicine into Medicare-specific programs. While the population covered by Medicare will benefit from access to virtual visits, this policy leaves those with private insurance, those covered by Medicaid, and uninsured patients out of the picture. Barriers also include reimbursement restrictions, licensure, credentialing restrictions, and broadband internet connectivity. Reimbursement and licensing restrictions have been temporarily suspended to allow greater telehealth access during the pandemic. This suspension highlights the need for sustainable change to increase access. The goal is to have telemedicine systems in place and health policies that support their use and availability so that a fully integrated and stable system is available, and patients are comfortable using it as a primary source of care.

### Access to Healthcare

Preexisting limitations of healthcare access were compounded during the COVID-19 pandemic by recommendations to avoid EDs and efforts to decrease outpatient visits for patients with noncommunicable diseases. Despite the increased availability of telehealth services, patients without internet access or smart devices cannot access this mode of healthcare effectively. Because of the racial inequity in COVID-19 cases and mortality, the need to address socioeconomic disparities affecting COVID-19 outcomes is clear, especially in New Orleans and other cities where preliminary data support this imbalance. To understand the breadth of the issue, the compilation of comprehensive nationwide data on the racial and socioeconomic breakdown of cases, complications, and deaths is imperative. These data can help guide population-specific interventions and health policy in preparation for future epidemics.

With the suspension of nonessential work in 43 states by mid-April, millions of Americans lost their jobs and health insurance.^68^ In some locations, 70% of individuals obtaining assistance from soup kitchens and food pantries had never visited one prior to the pandemic.^69^ Delays in business reopenings may lead to long-term repercussions in health and access to healthcare. In early April, the estimated unemployment rate was 13% (the highest rate of unemployment since the Great Depression), and 17 million Americans had applied for unemployment in the prior 4 weeks.^70^ Reduced access to care because of economic hardship increases the risk of disease, as classically, people of lower socioeconomic status typically have worse health outcomes than those in higher socioeconomic brackets. While incentives to protect the economy, such as lifting shelter-in-place mandates, may seem protective by increasing health insurance coverage, lifting isolation measures prematurely may risk a second wave of infection similar to the one that occurred during the 1918 Spanish flu epidemic and further deplete the primary care system.

The decrease in access to healthcare is also attributable to physician and medical staff shortage. The COVID-19 pandemic has amplified the stresses of global physician shortages.^71^ A 2019 report from the Association of American Medical Colleges identified a shortfall of 40,000 to 122,000 physicians during the prior decade.^72^ Older physicians are being called out of retirement, and medical students in their final year of study are accelerating their graduation to expand the workforce and aid in the increasing workload in response to the pandemic. As more healthcare workers are infected themselves, the population continues to age, and the ripple effects of COVID-19 impact the health of our nation, the burden on the healthcare system will continue to grow. The need for more physicians is undeniable, and this need must be reflected in the number of available residency positions. Facing such shortages, we cannot afford to turn away educated professionals seeking training.

### Correcting Disparities

In anticipation of New Orleans reopening, Ochsner Health, in conjunction with the City of New Orleans, began the first phase of a study to assess the COVID-19 disease burden in the Greater New Orleans area. Nontraditional testing centers were established, including one at the New Hope Baptist Church, to increase access to testing in communities where people lack transportation to travel to established testing sites.^73^ Selected study participants were tested for COVID-19 using both nasopharyngeal swabs and serum antibody tests. These data, combined with previously documented cases, will be used to gain insight into the extent of the spread of COVID-19 prior to the reopening of the city.^74^ Increasing the access to testing among underserved populations could facilitate quarantine measures and expedite connections to care prior to an escalation of symptoms.

In addition, outreach programs are targeting African Americans to help address healthcare disparities. The Louisiana Perinatal Quality Collaborative (LaPQC) has a dedicated health equity task force focused on addressing health inequity associated with provider practice.^75^ The LaPQC has implemented unit huddles that allocate time specifically for participating teams to discuss health equity in the context of COVID-19 and create space for the discussion of ideas to minimize the impact of implicit bias in practice.

Ochsner MedVantage clinics are creating healthcare access equity by removing financial barriers to care. These Ochsner-based clinics work primarily with patients who are considered high risk for hospital readmission and for poor outcomes with COVID-19. As a result, services have been focused on supporting high-risk populations via telemedicine with waived copay fees. When normal operations resume, MedVantage clinics will offer home visits and Lyft Health rides to patients who are unable to access transportation or who are physically unable to come to the clinic and will focus on addressing goals of care, including advance care planning.

Institutions and providers can take further action to tackle disparities in health by increasing awareness of implicit bias and how it can affect practice. Using tools such as those provided by the Government Alliance on Race and Equity can help identify and address racialized health inequities.^76^ Race-driven health disparities are prevalent throughout the American healthcare system and will require a unified response guided by comprehensive data to eliminate them.

## CONCLUSION

The word *global* in the term *global health* refers to the scope of current issues, not their location. Global health problems are too often portrayed as the distant issues of others—until they reach our doorstep. Globalization has made all populations vulnerable to disease, exploiting our interdependence with a clear predilection for the socioeconomically disadvantaged.

Innovative primary care solutions must address community inequity and the social determinants of health, clearly illustrated in Louisiana where African Americans account for the vastly disproportionate majority of COVID-19–associated cases and fatalities. Solutions should not be limited to the pandemic response but necessitate adaptation from the clinical to political level. During the pandemic, mental health has been unprioritized despite a rapidly increasing need for resources and initiatives that support collective resilience, the minimization of stigma, and the feeling of social closeness while physically distant. The unstable and unsupported telemedicine infrastructure has been forced to expand without proper patient or healthcare provider training, understanding of outcomes, or systems in place for dissemination or optimization. This infrastructure must become an integrated, fluent system prepared for disaster response, while also optimized for routine use to combat current barriers in primary care. Last, to aid in providing unified messages to government officials, systems must be developed to better synthesize medical knowledge among stakeholders. An increase in trained professionals is necessary, and the trained professionals and students at the crossroads of medicine, technology, economics, and diplomacy will be increasingly critical in the evolution of healthcare.

The digital age of the 21st century has played a role in many of the devastating effects of COVID-19. Yet technology has also provided and facilitated new tools, collaboration, and creativity for facing the challenges of this pandemic and challenges to come. Education, healthcare, and policy must reflect that potential.
